# Exploring the Acceptability of Web-Based Health Modalities in Individuals With Hypertension: Qualitative Study

**DOI:** 10.2196/72568

**Published:** 2025-08-25

**Authors:** Hillary Pelumi Banjo, Mark Stoutenberg, Lia K McNulty, Deja Spears, Lisa J Ware

**Affiliations:** 1 Department of Sport and Exercise Sciences, Durham University, Durham, United Kingdom; 2 Department of Health Behavior and Nutrition Sciences, University of Delaware, Newark, DE, United States; 3 Department of Kinesiology, Temple University, Philadelphia, PA, United States; 4 South African Medical Research Council/University of Witwatersrand Developmental Pathways to Health Research Unit, University of the Witwatersrand, Chris Hani Baragwanath Hospital, Soweto, Johannesburg, 2013, South Africa, 27 0117172831

**Keywords:** blood pressure, hypertension, South Africa, telemedicine, web-based health coaching

## Abstract

**Background:**

Hypertension is a significant public health concern in low- and middle-income countries, where access to care is crucial for effective treatment and control. Web-based health modalities provide a promising solution to overcome barriers to care, particularly in underresourced communities, if those communities engage with the technology.

**Objective:**

This study aims to examine the past experiences, perceptions, and preferences of using web-based health modalities for health care access among community members with or at high risk for hypertension.

**Methods:**

Semistructured interviews were completed with individuals randomly selected from a sample of community members in Soweto, South Africa, previously screened as having either elevated (systolic blood pressure [BP]≥120‐139 mm Hg or diastolic BP≥80‐89 mm Hg) or high (systolic BP≥140 mm Hg or diastolic BP≥90 mm Hg) BP to determine their past experiences using web-based services and what their perceptions were on using such services. An interview script, grounded in the Extended Unified Theory of Acceptance and Use of Technology (UTAUT2) model, was used to guide the interviews. Deductive thematic analysis was used to code the interviews and identify common themes.

**Results:**

A total of 178 community members (including 104 with elevated BP and 74 with high BP) were randomly selected and invited to participate in the study. Forty interviews were conducted with individuals from the elevated (n=20) and high (n=20) BP groups. Four major themes emerged from the interviews regarding using technology to receive health care services: (1) trust and credibility of health professionals in a web-based environment, (2) comfort level using technology to receive health care, (3) experience using technology to receive health care, and (4) preference for in-person versus web-based interactions.

**Conclusions:**

Despite being open and receptive toward the use of web-based health modalities to receive health care, participants preferred in-person interactions due to both a lack of experience using web-based health care and familiarity with traditional in-person health services. Further research is needed to understand how technology may aid future hypertension management efforts in urban African communities.

## Introduction

Hypertension, commonly referred to as high blood pressure (BP), is a major contributor to early mortality in Africa [1], accounting for 18 million fatalities globally in low- and middle-income countries (LMICs) [2]. Hypertension has become a significant public health concern in sub-Saharan Africa; in South Africa, prevalence estimates range from 27% to 58% [3,4]. Recent years have seen a marked rise in BP levels in LMICs, where a vast number of individuals are unaware that they have hypertension, and, in those who are aware of their condition, only 8% have their BP under control [5]. If left uncontrolled, hypertension can have fatal health consequences such as stroke, kidney failure, heart disease, and other cardiovascular issues [6]. The health burden from hypertension further widens gaps in health equity by disproportionately affecting marginalized populations while raising health care expenses for national health systems [7]. Access to primary care services plays a crucial role in achieving effective treatment and control of hypertension [8].

As the accessibility of health services for managing hypertension becomes increasingly important, exploring how technology can lower barriers to health care service delivery is imperative. One such promising solution is the use of eHealth technologies [9]. eHealth encompasses a broad spectrum of web-based health tools and modalities that leverage communication and information technologies to deliver health care services remotely [10]. By facilitating digital solutions, web-based health modalities have the capacity to enhance patient engagement, improve treatment adherence, and optimize health outcomes [11]. In the context of hypertension management, web-based health modalities offer a means to overcome barriers to access to care, particularly in underresourced communities that experience the greatest barriers to accessing health care services [12]. Web-based health modalities that may be useful in preventing and managing high BP in underresourced communities include web-based health coaching and telemedicine, both of which offer tailored care for individuals with varying BP levels.

For individuals with elevated BP (systolic BP≥120‐139 mm Hg or diastolic BP≥80‐89 mm Hg), behavioral changes play a pivotal role in managing their condition [13]. While medication may not be immediately necessary for this group, adopting lifestyle modifications, which include reducing salt and alcohol intake [14,15], is essential to preventing further elevation of BP. Web-based health coaching is an emerging eHealth modality that has demonstrated effectiveness in supporting health behavior changes in BP [16]. In Egypt, delivering health coaching, both in-person and via phone calls, considerably enhanced BP control and hypertension self-management activities among individuals in the intervention group [17]. The Community-Based Hypertension Improvement Project (ComHIP) in Ghana demonstrated that the use of web-based lifestyle modification in patients with hypertension significantly improved BP management, with control rates increasing from 42% to 72% [18]. Access to web-based health coaching can provide individuals with elevated BP in underresourced communities with tailored guidance and support for BP management.

For individuals with high BP (systolic BP≥140 mm Hg or diastolic BP≥90 mm Hg), obtaining medical care is crucial in managing their condition. Limited access to primary care services can hinder individuals with high BP from receiving timely diagnosis, antihypertensive medication, and monitoring, thereby impacting their ability to control hypertension effectively [19]. For patients with high BP, telemedicine—the remote diagnosis and treatment of patients using telecommunications technology—offers a viable solution to increase accessibility to primary care [20]. Improved access to care through telemedicine can significantly decrease the incidence of hypertension (mean decrease in systolic BP by −10 mm Hg over 5 years) with a simultaneous increase in BP control (from 44% to 62%) among treated patients. In Cameroon, engaging patients with high BP through telemedicine resulted in a significant decrease in their mean BP (decrease in diastolic BP by −15 mm Hg) compared to a control group [21]. By leveraging digital platforms and communication technologies, telemedicine transcends geographical barriers, allowing individuals in underresourced communities to receive personalized guidance and support in managing their BP levels [22].

While the technology for using eHealth as a solution to address barriers to accessing health care services is widely available in LMICs (eg, broad cell phone penetration) [23], there is limited information on how best to use these modalities, such as web-based health coaching and telemedicine, to increase accessibility. This study aims to address this gap by assessing individual community member’s past experiences, perceptions, and preferences of using web-based health modalities for BP management in an underresourced community in South Africa. Through this exploration, this study seeks to explore participants’ past experiences, current practices, and barriers. Additionally, this study seeks to inform strategies for enhancing health care access through web-based health modalities to improve outcomes for those with elevated and high BP in South Africa.

## Methods

### Overview of Study Methods

This study explored individual past experiences, perceptions, and preferences regarding the use of web-based health modalities to enhance access to health services for BP treatment and management. From July through September 2023, individuals who had been previously identified from home health screenings as having elevated or high BP were approached to gauge their interest in participating in the study. Semistructured interviews were conducted with a randomly selected sample of eligible community members using an interview script adapted from the Extended Unified Theory of Acceptance and Use of Technology (UTAUT2) model.

### Setting and Home Health Screenings

This study took place in Soweto, a conglomerate of 29 townships that is home to more than 1.6 million individuals, situated to the southwest of Johannesburg, South Africa. This study builds upon previous work in which home health screenings were conducted with a large sample of community members in Soweto (n=2041). The home health screenings were conducted by community health workers (CHWs) [24], in which eligible household members provided their informed consent, demographic data (ethnicity and years of education), and anthropometry (height), and completed a health questionnaire on their medical history and health behaviors, including tobacco and alcohol usage, and also completed the Physical Activity Vital Sign questionnaire to assess physical activity levels [25]. BP measurements were taken according to the International Society of Hypertension guidelines, with participants categorized as having either elevated (systolic BP≥120‐139 mm Hg or diastolic BP≥80‐89 mm Hg) or high (systolic BP≥140 mm Hg or diastolic BP≥90 mm Hg) BP.

### Study Participant Selection and Recruitment

Participants for this study were randomly selected from 2 lists of individuals with elevated and high BP, respectively, who were a part of the CHWs’ home health screenings in May 2023 and had consented to being contacted for future studies. Two research assistants from the Wits Health HUBB [26] visited the homes of the randomly selected individuals in Soweto in July 2023 to conduct semistructured interviews. Standard operating procedures involved attempting to locate participants at the addresses provided during the initial home health screening, and in cases where participants could not be found at the specified address, attempts were then made to contact them using the phone numbers they had previously provided. As a final effort to establish contact, the research assistants visited participant homes for a second time. If a potential participant was unreachable or declined to participate, additional individuals were randomly selected from each respective participant list. This process was repeated until interviews were conducted with 40 participants (20 with elevated BP and 20 with high BP).

Efforts were made to ensure gender diversity in the sample; however, there was a sex skew due to participant availability and willingness. The final sample generally reflected the demographic composition of Soweto, which is predominantly made up of Black South Africans, many of whom experience socioeconomic challenges that affect their access to health care.

### The UTAUT2 Model

The UTAUT2 is an extended model designed by Venkatesh et al [27] to comprehend individuals’ attitudes and behaviors toward novel technologies. It extends the original framework (UTAUT), which focused on organizational settings and included performance expectancy, effort expectancy, social influence, and facilitating conditions, by incorporating hedonic motivation, price value, and habit to make it more suitable for consumer technology use contexts [27]. The UTAUT2 model aims to depict how different factors influence technology acceptance and usage behavior across diverse populations and contexts. In this study, the UTAUT2 served as a conceptual framework for exploring the acceptability of web-based health modalities among community members in an underresourced South African context. While the UTUAT2 was originally designed to assess actual technology use [27], it was deemed suitable for use in this study despite participants having little to no prior experience with web-based health modalities. This decision was informed by Ong et al [28] and their adaptation of the UTAUT2 to explore the acceptance of telemedicine in a similar LMIC context. The Ong [28] adaptation involved the inclusion of factors such as personal experience, geographical location, and data privacy issues to capture personal, environmental, and situational factors influencing participants’ technology acceptance. These modifications allow for a more precise exploration of potential facilitators and barriers to using web-based health care technologies in low-resource, real-world contexts.

### Development of Interview Guide

The semistructured interview guide was developed by mapping questions to the UTAUT2 model (see Multimedia Appendix 1). The initial section of the interview guide collected data on participants’ age, sex, smartphone ownership, and internet skills. Subsequent sections of the interview guide encompassed 9 factors: personal experience, geographical location, data privacy concerns, social influence, effort expectancy, performance expectancy, hedonic motivation, usage behavior, and intention to use eHealth technology. To ensure the suitability and accessibility of the interview guide for community members, a rigorous translation process was undertaken, involving two members of the research team from the local community, in which one member translated the guide to isiZulu, while the other subsequently back-translated the guide to English.

### Individual Interviews With Community Members

The interviews took place between July and September 2023 in the homes of the participants and were recorded using an audio recorder by the interviewer. Interviews were conducted in the participant’s preferred language and lasted between 12 and 20 minutes.

### Ethical Considerations

The procedures and materials of this study were approved by the Human Research Ethics Committee (Medical) of the University of Witwatersrand (ref. M200941 and M170334). Participants in this study received an information sheet along with an oral description of the study, which outlined the study procedures as well as associated risks and benefits. Informed consent was obtained from all participants prior to engaging in the study through a signed consent form. All participant responses and data were deidentified to protect their privacy. As compensation reimbursement for their time and effort, participants received R50 (approximately US $2.73) upon completion of the interview.

### Quantitative Analyses

Independent sample *t* tests were conducted using SPSS software (version 29.0; IBM Corp) to assess differences between participants with elevated and high BP based on their age, smartphone ownership, internet skills, average age of smartphone ownership, and gender. Statistical significance was determined at *P*<.05. Additionally, chi-square tests of independence were performed to examine group differences in level of education, self-reported medical history (including prior hypertension diagnosis, current treatment, diabetes, and previous stroke), and behavioral health factors. The Fisher exact test was used when expected cell counts fell below suitable thresholds.

### Qualitative Analyses

Individual interviews were analyzed using a deductive thematic approach, drawing on the UTAUT2 as the guiding framework. The thematic analysis process was guided by Braun and Clarke’s 6-phase framework for thematic analysis, which comprises data familiarization, initial code generation, search for themes, reviewing of themes, defining and naming themes, and final report production [29]. A preliminary codebook was produced based on 8 factors of the UTAUT2: effort expectancy, performance expectancy, hedonic motivation, geographical location, personal experience, data privacy concerns, social influence, and intention to use. Two transcripts were then coded by 2 members of the research team, who iteratively refined the codebook as more transcripts were reviewed. All interview transcripts were uploaded to Dedoose (SocioCultural Research Consultants, version 7.0.23) for coding.

The coded data were reviewed and discussed by the study team several times to identify significant patterns and variations across categories and between the two BP groups. The analysis concentrated on the areas in which participants provided the most significant and thorough responses, resulting in the emergence of themes that better characterized the core concerns, experiences, and perceptions expressed throughout the interviews. Although data saturation was not the primary goal of this study due to its explanatory, time-sensitive nature, we sought to obtain as much rich, relevant data from participants as possible shortly after their home screenings. We identified repetition in participant responses to interview questions, indicating that a variety of perspectives had been adequately captured. This is consistent with Hennink and Kaiser’s [30] broader definition of data saturation, which highlights the point in data collection when no new themes arise and additional data becomes redundant. Reflexivity was applied throughout the analysis process as the research team held periodic meetings to discuss how our assumptions may have influenced the analysis and interpretation of participant responses [31]. Collaborative coding and iterative discussions within the research team also contributed to credibility [32], ensuring that interpretations were based on participants’ narratives.

## Results

### Overview

**Figure 1.**
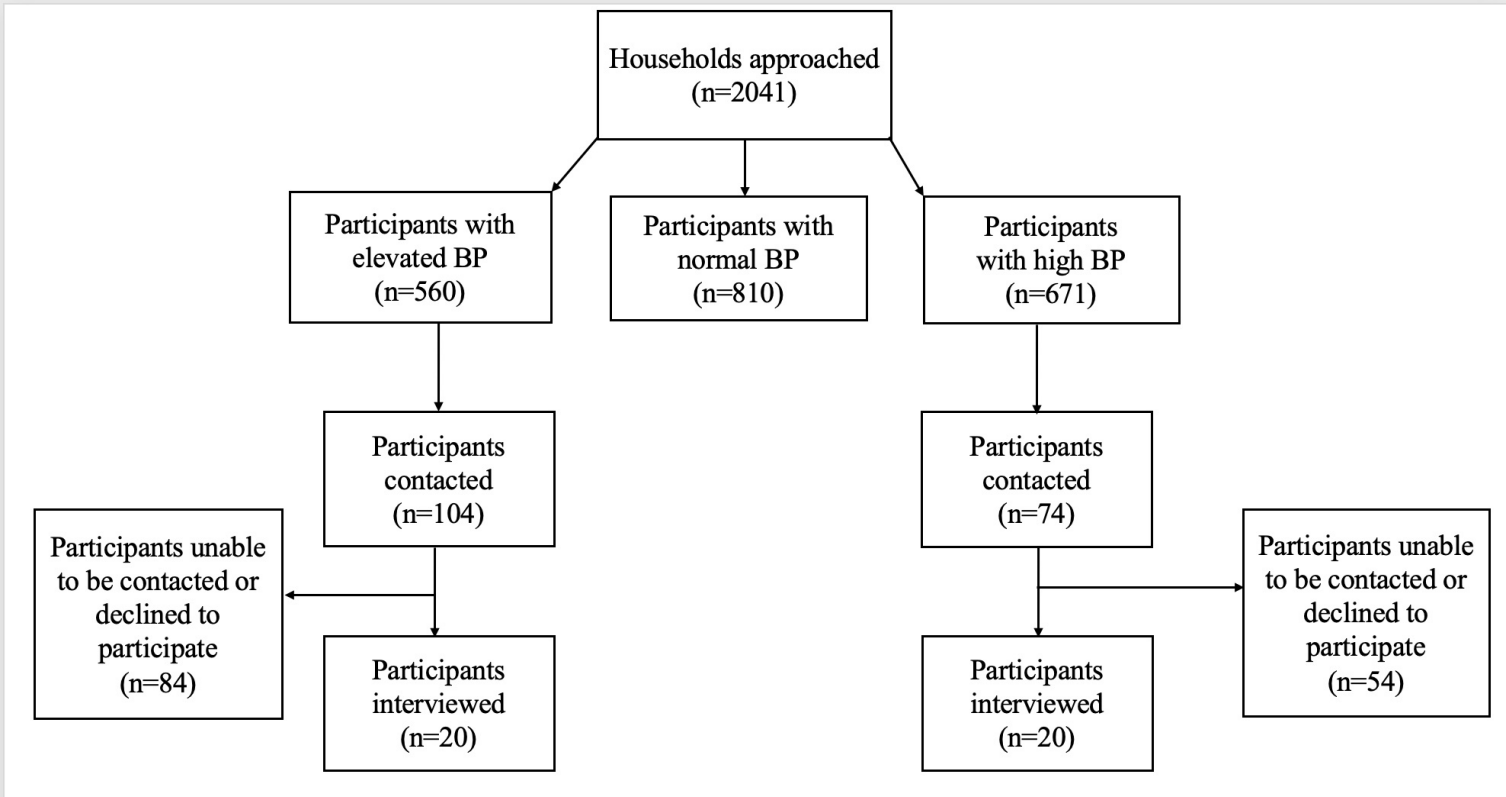
Flow diagram of participant identification, selection, and individual interview recruitment. BP: blood pressure.

During the home health screenings, CHWs visited 2041 community households (Figure 1), of which 560 and 671 individuals were screened as having elevated and high BP, respectively. A target sample of 40 interviews was set, requiring the random selection and invitation of 178 community members (104 with elevated BP and 74 with high BP) to participate in the study. The final interview sample comprised 40 participants (12 men and 28 women) between 27 and 84 years of age (Table 1). Based on data collected from the original home health screenings, all participants self-identified as Black, and nearly three-quarters (29/40, 72%) reported having 7‐12 years of education. Approximately half of the participants (21/40, 52%) reported daily alcohol consumption, and more than half (26/40, 65%) did not meet the recommended 150 minutes per week of physical activity. Significant differences were observed between the elevated and high BP groups in terms of their average age of smartphone ownership and internet skills, and gender.

**Table 1.** Characteristics of individuals with elevated and high BP^a^ completing the individual interviews (N=40).

Variable	Individuals with elevated BP (n=20)	Individuals with high BP (n=20)	Overall sample (N=40)	*P* value
Age (years),^b^ mean (range)	53.3 (27-81)	53.7 (33-84)	53.5 (27-84)	.99
Own smartphone,^b^ n (%)				.50
Yes	13 (65)	15 (75)	28 (70)	
No	7 (35)	5 (25)	12 (30)	
Average age (years), mean (range)				.004^c^
Own smartphone	49.8 (27-68)	49.0 (33-67)	49.4 (27-68)	
Does not own smartphone	59.8 (36-81)	71.2 (57-84)	64.0 (36-84)	
Internet skills,^b^ n (%)				.75
Yes	7 (35)	8 (40)	15 (38)	
No	13 (65)	12 (60)	25 (62)	
Average age (years), mean (range)				.008^c^
With internet skills	47.2 (27-73)	45.2 (33-60)	46.2 (27-73)	
Without internet skills	56.6 (31-81)	59.9 (44-84)	58.1 (31-84)	
Sex,^d^ n (%)				.04^c^
Female	17 (85)	11 (55)	28 (70)	
Male	3 (15)	9 (45)	12 (30)	
Self-reported ethnicity,^d^ n (%)				
Black African	20 (100)	20 (100)	40 (100)	
Education (years),^d^ n (%)				.91
1‐6	2 (10)	2 (10)	4 (10)	
7‐12	15 (75)	14 (70)	29 (72)	
>12	3 (15)	4 (20)	7 (18)	
Anthropometry,^d^ mean (range)				
Height (cm)	141.8 (107-170)	137.5 (135-180)	139.6 (107-180)	
Self-reported medical history,^d^ n (%)				
Hypertension prior to diagnosis	10 (50)	7 (35)	17 (43)	.52
On treatment	10 (50)	7 (35)	17 (43)	.52
Diabetes mellitus	1 (5)	0 (0)	1 (3)	>.99
Previous stroke	0 (0)	1 (5)	1 (3)	>.99
Behavioral health factors^d^				
Tobacco use, n (%)				.76
Current use	1 (5)	2 (10)	3 (8)	
Past use	3 (15)	2 (10)	5 (13)	
Never used	16 (80)	16 (80)	32 (80)	
Alcohol consumption, n (%)				.60
Daily	12 (60)	9 (45)	21 (52)	
1‐6 times per week	4 (20)	6 (30)	10 (25)	
1‐3 times per month	2 (10)	4 (20)	6 (15)	
Never or rarely	2 (10)	1 (5)	3 (8)	
Physical activity <150 min/week, n (%)	13 (65)	13 (65)	26 (65)	>.99

a BP: blood pressure.

b Indicates that the data were collected during the individual interviews.

c Statistically significant difference (*P*<.05) between participants with elevated and high BP.

d Indicates that the data were collected during the home health screenings.

From the 40 interviews, responses to 9 Likert-type questions using a 5-point Likert scale were excluded due to a high number of nonnumeric and irrelevant responses. In 143 cases, participants did not provide a numerical value but rather a general response to the question (eg, “No, it’s not enough, because it ends before you expected it to,” “It lasts maybe 2 weeks,” and “In my opinion, I would rather go to the clinic”). In 23 cases, participants either did not provide a response relevant to the question or did not answer the question. Due to these inconsistencies, these data were excluded from the final analysis and not reported. See Multimedia Appendix 2 for a breakdown of missing data for each of these questions.

Analysis of the open-ended interview questions involved the development of codes based on the 8 UTAUT2 factors. Participants did not engage with all of them equally; some were mentioned briefly (time of seeking advice and technical difficulty in their personal experience using technology) or not at all (knowledge of others using health coaching, knowledge of others using telemedicine, others’ description of health education experience, awareness of negative beliefs, and description of negative beliefs relating to the code social influence). Based on the codes discussed most frequently by participants, four main themes emerged regarding participants’ perspectives and experiences about using technology for health care: (1) trust and credibility of health professionals in a web-based environment, (2) comfort level using technology to receive health care, (3) experience using technology to receive health care, and (4) preference for in-person versus web-based interactions. Three themes (comfort level using technology to receive health care, experience using technology to receive health care, and preference for in-person versus web-based interactions) directly mapped to 4 factors of the UTAUT2—personal experience, effort expectancy, facilitating conditions, and intention to use. With the theme of trust and credibility of health professionals in a web-based environment, mapping indirectly to the UTAUT2 factor performance expectancy. A sample of quotes supporting each theme can be found in Table 2.

**Table 2.** Emergent qualitative themes mapped to the UTAUT2^a^ model with illustrative quotes from the individual interviews.^b^

Theme	Mapping to the UTAUT2 model	Examples of illustrative quotes representing the emerging themes
Trust and credibility of health professionals in a web-based environment	(Performance expectancy)	“I would be happy for it in a way, yes, I would be happy for it because there are things that we don’t know right? (I: Yes.) things that we don’t have information on. There is a person who can maybe, when I have certain questions...yes, I will be happy for it.” “Eh, most of us people. We are much open to strangers than to a person you are close to. Yes, because with a stranger you can talk about anything. And then you won’t hear it anywhere. Except where you left it. I don’t know if it makes sense.” “I am one person who is open, and I believe that if I share, I will find things that will help me, but also it depends on the environment that you are in and the people around you, you understand? But if it’s a professional I don’t mind, because if you don’t tell them certain things how will they help you? So, if I keep information away from them...they deal with this on a daily basis, so it’s a person who has studied for this so that they can help me, so I will be open.” “No, as long as it’s the doctor, it’s the doctor that has to heal us. (I: Mm.) So, I cannot be afraid of the Doctor. (I: Mm.) When you’re a person, some things you can’t say, but when it’s the doctor. I need to be open for them because they are the one examining, they are helping, so what would I be hiding?”
Comfort level using technology to receive health care	Hedonic motivation	“I would really be happy for that, as I was saying, right now I have a problem with my grandma, So, I don’t know which diet to start with because of the age she is in. Even I, myself, I am that person who likes to lose weight, control my weight but I think I control it in a wrong way, so if there is someone who can give me that diet, I would really appreciate it.”
	Effort expectancy	“Yes, that would be easy because days are not the same sometimes you get tired then you request for a video call from them and things.” “I think the video call one, because with the internet someone could hack it and you find that your information is spread. (I: Yes.) You see, at least a video call is private, because I know with this one, we call then it’s done. (I: Yes.) It doesn’t remain on the phone. (I: Mm.) Yes.” “Like I said I would talk because, I don’t know the nurse and they don’t know me so they can help me (I: Hmm) and I would be happy to receive help from the nurse.” “No, it won’t be difficult the thing is, I don’t have their contacts details, I haven’t connected with them. But the thing is I am not working and maybe those things need money, you understand...Maybe that’s where the difficulty can be, but if I do have the money, there are a lot of things we can do on the phone.”
	Facilitating conditions	“The problem is airtime *exclaims* it wouldn’t be hard when it comes to sending a message on WhatsApp and all of that, but airtime brother no...but then financially there is no money. My kids even know that they should call me, because I don’t have money.” “I think the internet would be ideal as I said my challenge is airtime...I don’t have money, the resources...the resources...”
Experience using technology to receive health care	Personal experience	“No, I talk to them face-to-face.” “No, I go to the clinic.” “I think connecting online is the best than going there...” “I called the clinic when I lost my child, so I called the ambulance.”
Preferences for in-person versus web-based interaction	Intention to use	“It is better I see them. Face to face. So they see as well. That this person is sick. Fine. They can see that I’m unable to speak. Yeah?” “I would like to go to him, maybe someday you find that I don’t have the money to load airtime; it is better I go see him personally.” “I would rather go to the clinic. Because I would not be able to trust the person on the phone, while going to the clinic I will be assisted by doctors, I know they will be able to tell me what is wrong with me.” “No, I prefer going to the clinic. (I: Why?) At the clinic they will be able to assess me properly and if there is anything wrong, they will be able to transfer me to the doctor. If I make a phone call, they will not be able to properly diagnose me. At the clinic they make test and find out what is wrong.”

a UTAUT2: Extended Unified Theory of Acceptance and Use of Technology.

b () indicates an indirect mapping to the UTAUT2 model.

### Trust and Credibility of Health Professionals in a Web-Based Environment

Participants expressed a general openness to engaging with health professionals remotely, emphasizing that as long as the information provided contributes to improving their health, they would be receptive to such interactions.

A majority of those with elevated BP reported being pleased to remotely receive information about their health. These individuals emphasized that regular access to web-based health services would be highly beneficial, and they would be eager to obtain medical advice to enhance their health. One interviewee commented:

*I would be happy for it in a way, yes, I would be happy for it because there are things that we don’t know right? Things that we don’t have information on. There is a person who can maybe, when I have certain questions...yes, I will be happy for it*.

Similarly, most participants with high BP indicated that they would feel comfortable speaking remotely with a health professional, particularly if it is a doctor. An interviewee commented:

*No, as long as it’s the doctor. It’s the doctor that has to heal us so I cannot be afraid of the doctor. When you’re a person, some things you can’t say, but when it’s the doctor I need to be open for them because they are the one examining, they are helping, so what would I be hiding?*

These individuals noted that they would be willing to engage in web-based consultations as this approach could be lifesaving and offer a more efficient means of receiving health care compared to the slower rate of receiving health services typically experienced in-person at clinics.

### Comfort Level Using Technology to Receive Health Care

While age differences and associated levels of internet proficiency between participants in the elevated and high BP groups may have influenced the reporting of comfort levels in using technology for health care services (Table 1), most participants reported feeling comfortable with the idea of receiving health care via phone and the internet, regardless of their BP status. This was generally attributed to the ability to access assistance for their health needs through these modalities. Participants with elevated BP expressed comfort using technology for health care services, particularly through the phone and internet, as it offered an opportunity to obtain valuable health-related information. Some participants noted their comfort stemmed from a need for dietary guidance for both them and their families. One interviewee remarked:

*I would be free. The thing is I want to know because my cousin had diabetes, and they gave her some things. They showed her how to eat, to buy what kind of things, veggies. Yes. I would be free because I want knowledge.*

Participants also indicated that they would be comfortable using technology to obtain health-related knowledge and improve their health, and would appreciate the convenience of being able to make calls and speak with health professionals via the internet.

Participants with high BP similarly reported comfort using technology for health care, particularly valuing the privacy that video calls offer for discussing confidential health issues. One participant noted:

*I think the video call one, because with the internet someone could hack it and you find that your information is spread. At least a video call is private, because I know with this one, we call then it’s done. It doesn’t remain on the phone.*

Moreover, participants expressed that technology provides a platform for anonymously discussing their health concerns, which was reflected in another participant’s comment:

*Like I said, I would talk because I don’t know the nurse and they don’t know me, so they can help me, and I would be happy to receive help from the nurse.*

The availability of resources for using technology to receive web-based health care appeared to influence participants’ comfort levels in using technology for health care services. Some participants reported that financial constraints, specifically the inability to afford airtime and data, could hinder their ability to comfortably engage with health professionals remotely. One interviewee remarked:

*The problem is airtime. It wouldn’t be hard when it comes to sending a message on WhatsApp and all of that, but airtime brother no...but then financially there is no money. My kids even know that they should not call me, because I don’t have money.*

### Experience Using Technology to Receive Health Care

Although not explicitly asked, nearly all participants reported having no prior experience using technology to receive health care. Participants with elevated BP generally indicated a lack of experience with telehealth services. Similarly, most participants with high BP reported no experience using technology for health care, as they were accustomed to physically visiting clinics. This is reflected in statements such as, “No, I talk to them face-to-face” and “No, I go to the clinic.” Most participants did not know of anyone who used technology to receive health care services, attributing this to the community’s reliance on traditional, face-to-face health care interactions. This is evident in the comment by a participant:

*There is no one that I know, the old women I know physically go, we live with them around here and sometimes we meet at the clinic or the gym.*

For the few participants who reported previous experience with technology in a health care context, it was typically using the phone to request emergency services, such as an ambulance, or contacting a pharmacy for medication.

### Preferences for In-Person vs Web-Based Interaction

Most participants across both the elevated and high BP groups reported a preference for in-person interactions over web-based consultations. Participants in the elevated BP group emphasized that in-person interactions allowed for more effective communication of symptoms and the receipt of accurate diagnoses. As one interviewee noted:

*It is better I see them face to face. So they can see as well, that this person is sick. They can see that I’m unable to speak. Yeah?*

Additionally, some participants noted that in-person interactions allowed them to access health care services without the financial burden associated with web-based consultations, such as the cost of data or airtime. One participant remarked:

*I would like to go to him. Maybe someday you find that I don’t have the money to load airtime; it is better I go see him personally*.

While some participants in the high BP group liked the anonymity of web-based appointments, other participants reported a greater preference for in-person interactions, citing concerns about trust and the reliability of web-based consultations. One interviewee commented:

*I would rather go to the clinic because I would not be able to trust the person on the phone, while going to the clinic I know I will be assisted by doctors who can tell me what is wrong with me*.

Furthermore, participants felt that in-person visits allowed for a more comprehensive diagnosis compared to web-based consultations, where certain issues might be overlooked. This sentiment is reflected in the comment:

*No, I prefer going to the clinic. At the clinic, they will be able to assess me properly, and if there is anything wrong, they will be able to transfer me to the doctor. If I make a phone call, they will not be able to properly diagnose me. At the clinic, they make tests and find out what is wrong.*

## Discussion

### Principal Findings

This study explored the past experiences, perceptions, and preferences of community members with or at high risk of hypertension regarding the use of web-based health modalities to enhance access to health services for BP treatment and management. We found that participants expressed trust and credibility in health professionals within a web-based environment. Participants also expressed comfort using technology to receive health care. However, when presented with an option between in-person and web-based consultations, participants reported a preference for in-person consultations. Importantly, we found no differences in the responses between individuals in the elevated and high BP groups.

### Comparison to Prior Work

The theme of trust and credibility of health professionals in a web-based environment reflects our participants’ openness and willingness to engage with web-based modalities for receiving health services, which aligns with findings from other studies conducted in similar settings [33]. Similar to studies conducted in Bangladesh and Saudi Arabia, older adults with cardiovascular disorders expressed favorable perceptions and a willingness to engage with telemedicine for health care services [34,35]. While these findings do not directly map onto any of the original validated UTAUT2 factors, echoing the criticisms about the model’s lack of applicability within health care settings [36], they highlight the role of trust in shaping the feasibility and acceptance of novel technologies within underresourced contexts. These findings support the inclusion of trust as an additional factor when adapting the UTAUT2, as done by Schomakers et al [37], who suggested that trust is especially important in uncertain situations; the degree of trust is influenced by participants’ perceptions of the technology’s reliability, predictability, and their experience with it [38]. Arguably, these findings also map indirectly to the factor of performance expectancy, defined as the degree to which individuals believe the use of a specific technology would help them obtain a desired outcome [27]. Our participants emphasized that they would be receptive to using web-based modalities to receive health care, provided the information and guidance they receive is from a credible source (eg, trusted health professionals) and would improve their health. This underscores the pertinent role of health professionals in shaping whether or not participants choose to engage with web-based modalities to receive health care.

Despite this openness, participants in our study highlighted a notable lack of experience with web-based health modalities, such as telemedicine and web-based health coaching. This observation is consistent with findings from work in Ethiopia involving adults with cardiovascular, endocrine, and respiratory disorders, in which participants expressed enthusiasm for web-based health services, but had minimal to no prior exposure to using such technologies [33]. These findings emphasize the relevance of the factor of personal experience from UTAUT2 [27], which plays a crucial role in shaping user acceptance and engagement with technology [28]. Despite South Africa’s high mobile phone adoption rate [39], severe digital gaps continue, notably in terms of internet access. According to national data, only 1.7% of rural families and 17.3% of urban households have home internet access [40]. Soweto, while being classified as an urban area, shares many traits with many underresourced communities, such as unreliable infrastructure and low digital literacy. These systemic barriers contribute to participants’ limited exposure to and engagement with web-based health services, despite their interest.

The UTAUT2 factor of effort expectancy, or the perceived ease of using web-based health modalities [27], is also a relevant factor shaping participant perceptions. A majority of participants reported having no internet skills, challenges with limited data, and an inability to purchase airtime—issues that impede consistent and comfortable use of web-based modalities to receive health care. This is in line with the findings of a study conducted in Saudi Arabia, where effort expectancy positively predicted patients’ adoption of an eHealth system [41], suggesting that additional interventions may be needed to build familiarity and confidence. This may include introductory training sessions or user-friendly platforms designed to bridge the gap between willingness and practical use, particularly in LMIC contexts [42].

Despite an openness and willingness to receive health services remotely, our participants still preferred in-person over web-based interactions, mostly due to concerns about the reliability of web-based interactions. This finding is in line with that of Moulaei et al [43], who discovered that individuals expressed a preference for in-person interactions due to reliability, familiarity, and concerns that web-based interactions might overlook crucial diagnostic details of one’s medical condition that could be observed through direct observation and testing, leading to a less thorough assessment of their health [44]. These findings reflect the UTAUT2 factor of hedonic motivation, the anticipated satisfaction derived from using technology [27]. While participants reported comfort with the idea of web-based consultations, the majority preferred in-person consultations due to higher trust, familiarity, and perceived effectiveness. This suggests that, while there may be some hedonic motivation, it is limited by other issues, such as trust and communication difficulties in web-based environments.

Furthermore, these findings map to the UTAUT2 factor of intention to use, which relates to an individual’s likelihood to adopt a specific technology [27]. Although participants in our study expressed an interest in web-based health modalities, their preference for in-person consultations indicates a hesitance toward the adoption of web-based health modalities. While studies have also shown that web-based consultations can produce comparable results to in-person consultations [45,46], educating patients on these findings through targeted awareness campaigns, workshops, and easily accessible informational materials could help build trust in web-based modalities [47]. Additionally, introducing web-based sessions into routine care as a supplement, rather than a substitute, may gradually improve familiarity and trust and pave the way for wider acceptance of web-based health solutions [48].

The facilitating conditions factor within the UTAUT2 model provides a useful lens to understand how age influences preferences for web-based health services. While cell phone ownership is widespread in LMICs, including settings like Soweto [39], older adults often face unique challenges that limit their ability to effectively use digital health technologies [45]. These challenges include a lack of confidence, minimal exposure to technology, and difficulty navigating digital platforms [49,50]. In contrast, younger participants may benefit from more supportive facilitating conditions, such as greater familiarity with technology, prior experience with web-based platforms, and access to digital literacy resources [51]. This disparity highlights the critical role of facilitating conditions, which include access to supportive infrastructure, training resources, and user-friendly technologies, encouraging older adults to use web-based health modalities. Without addressing these age-related barriers, older adults in LMICs may continue to face difficulties engaging with web-based health services, despite their willingness and openness to using such modalities.

The absence of discussion around several UTAUT2 factors (eg, social influence, geographical location, and data privacy concerns) is notable given that while some participants noted the benefit of avoiding clinic visits, others were more concerned with issues relating to access and experience adopting web-based modalities. Similarly, social influence (family, friends, or other community members) appeared to have little impact on participants’ perceptions toward web-based health modalities. Geographical location and data privacy concerns, which are important contextual factors highlighted by Ong et al [28], were not discussed by our participants. This may reflect the relatively novel nature of web-based health modalities in this context, making them less likely to be part of shared experiences and issues of concern during social discussions [52].

### Limitations and Strengths

There are two main limitations to this study. First, participants were asked about their willingness to engage with web-based health modalities, with which most had no experience. Although willingness to use eHealth services offers insightful information for future work, actual usage behavior often differs in real-world settings [53]. In the future, to mitigate this limitation, prescreening of potential participants may help balance those who have and do not have experience with web-based health modalities to strike a better balance. Second, this study had a sex imbalance with lower male representation [54], potentially limiting the generalizability of the results. While our randomization process ensured greater generalizability to the community as a whole, it did not address potential sex imbalances. To mitigate this in the future, the randomization could be stratified based on sex to ensure balanced representation.

At the same time, this study has several strengths that enhance the reliability and relevance of its findings. First among these is the focus on capturing the voices of individuals with lived experience who recently underwent a home health care screening, were diagnosed with elevated or high BP in an underresourced LMIC community, and needed a lifestyle intervention or clinical follow-up. Second, the inclusion of participants with lived experience enhances the findings by providing a more nuanced perspective of the barriers and opportunities associated with web-based health modalities. Third, the rigorous randomization process ensured that participant selection was unbiased, thereby strengthening the validity and generalizability of the study’s findings across the targeted population. Finally, using the UTAUT2 model as a foundation for the study provides a structured approach to analyzing participants’ perceptions, allowing for a deeper understanding of the complexities surrounding the use of web-based health services for individuals with elevated and high BP.

### Future Directions

The findings of this study highlight the need to address technological constraints when developing and implementing eHealth solutions in similar settings. Hence, including web-based sessions into routine clinical consultations as a supplement may gradually improve trust and familiarity in the adoption of web-based health services. Additionally, the findings of this study will support efforts to expand access to health care for community members by offering options, including web-based health modalities for receiving care.

### Conclusions

This study explored the past experiences, perceptions, and preferences of community members in an underresourced South African community on the use of web-based health modalities for BP management. A qualitative, thematic analysis of semistructured interviews revealed that, while participants were generally open to using web-based modalities for receiving health care, their preferences tended toward traditional in-person care. This preference was influenced by factors such as limited prior experience with web-based health modalities, trust and reliability of web-based consultations, effective symptom communication, and greater familiarity with traditional in-person health care. These findings demonstrate how trust and familiarity influence the acceptability of web-based health care within this context.

## Supplementary material

10.2196/72568 Multimedia Appendix 1 Individual interview guides for patients with elevated and high blood pressure.

10.2196/72568 Multimedia Appendix 2 Breakdown of nonnumeric, unanswered, and irrelevant responses that were excluded from the final analysis.

## Abbreviations

BP: blood pressure
CHW: community health worker
ComHIP: Community-Based Hypertension Improvement Project
LMICs: low- and middle-income countries
UTAUT2: Extended Unified Theory of Acceptance and Use of Technology
