# Supplementary material for: Exploring the Acceptability of Web-Based Health Modalities in Individuals With Hypertension: Qualitative Study
Source: J Med Internet Res. 2025 Aug 25;27:e72568. doi: 10.2196/72568 (PMC12377872; doi:10.2196/72568)
Supplement: Multimedia Appendix 1 [file jmir-v27-e72568-s001.docx]

**Technology - Individual Interview Guide for Patients with Elevated Blood Pressure**

USE: This individual interview guide will seek out the thoughts and feelings of patients with elevated BP (based on their BP measurements during an earlier home-based screening) on being referred to and accessing an online health educator to provide them with health support. This survey uses the extended Unified Theory of Acceptance and Use of Technology (UTAUT2) model to guide the development of the following question guide.

Today, during this conversation, I would like to ask you a series of questions about how you use your telephone and the internet and your thoughts about receiving health education through either your phone or your computer. Some of the questions will refer to receiving health education by speaking to a professional health educator over the phone about nutrition, physical activity, and healthy lifestyles.

Demographic Questions

- May I please know how old you are?
- What do you identify as, male, female, or other?

mHealth Questions

1. Do you have a smartphone?

*yes no*

If participant answered no skip to question #6.

- 1. What do you use your phone for? (select all that apply)

|  | Texting |
| --- | --- |
|  | Calling |
|  | Video calling |
|  | Taking/storing photos |
|  | Using the internet |
|  | Sending and receiving emails |
|  | Using apps |
|  | Watching videos |
|  | Playing/storing music |

1. How much data do you feel you have monthly for your typical technology use?

| **1** | **2** | **3** | **4** | **5** |
| --- | --- | --- | --- | --- |
| *More than  enough data* |  |  |  | *Not Enough  Data* |

1. How often do you use apps on your smartphone?

| **1** | **2** | **3** | **4** | **5** |
| --- | --- | --- | --- | --- |
| *Multiple times  a day* | *Once a day* | *Every couple  of days* | *Once a week* | *Never* |

1. If the participant answered **never to #3,** *What are the reasons why you do not use apps? (select all that apply).*

|  | Don’t have the time |
| --- | --- |
|  | Too expensive |
|  | Don’t like apps |
|  | Don’t want to use apps |
|  | Don’t know how to use apps |
|  | Not enough data |
|  | Other (please specify): __________________ |

1. Have you ever used your phone or internet to access a health educator (speak to a professional over the phone or internet about nutrition, physical activity, and healthy lifestyles)?

*yes no*

- 1. (If they answered **yes**) How would you rate the quality of the phone call on a scale from 1 to 5?

| **1** | **2** | **3** | **4** | **5** |
| --- | --- | --- | --- | --- |
| *Poor* |  | *Average* |  | *Excellent* |

**Personal Experience**

1. Do you have experience receiving any type of health services through your phone (such as an app) or the internet?

If NO, skip to the next section. If YES, go to question #7

1. Could you tell me a little about this experience?
   1. For what health condition did you receive health services through your phone or the internet?
   2. How long ago was this?
   3. How would you describe your overall experience?
   4. Did you experience any technical difficulties that affected the quality of care you received from your telemedicine service?

**Geographical Location**

1. How strong is the internet connection where you live?

| **1** | **2** | **3** | **4** | **5** |
| --- | --- | --- | --- | --- |
| *Poor* |  | *Average* |  | *Excellent* |

1. Do you feel like you have local health and wellness resources (e.g., gym) nearby?
   1. How does the distance to health and wellness resources in your community impact your decision to go there?
   2. How does the distance to health and wellness resources impact your willingness to try receiving health guidance through your telephone or the internet?

**Data Privacy Issues**

1. How do you feel about talking to a health educator who you do not know on your phone or through the internet?
2. How do you feel about the safety of the information you provide to a health educator through your phone or the internet?
   1. How might this impact your willingness to share your health information?

**Social Influence**

1. Do you know or have you heard of anyone who has received health education from a professional health educator through their phone or the internet?

If NO, skip to the next section. If YES, continue to question #13.

1. How did they describe their experience?
2. Do you know of any negative beliefs attached to accessing a virtual health educator? Could you please describe them?

**Effort Expectancy**

1. How hard do you think using your phone to access a health educator will be?
2. How do you feel about your ability and skills to use the internet?
3. How do you feel about your ability to navigate through the internet to access a health educator?
   1. Such as scheduling appointments, turning on your camera and audio, and navigating through software?

**Performance Expectancy**

1. How easy would it be to use your phone or the internet to access a health educator whenever you want?

**Hedonic Motivation**

1. How comfortable would you feel receiving health education from a professional health educator through the internet?
   1. Probe further and ask how/why.

**Usage Behavior**

1. How likely are you to access a health educator through the internet to communicate your health concerns and improve your health? Why?

| **1** | **2** | **3** | **4** | **5** |
| --- | --- | --- | --- | --- |
| *Not very likely* | *Not likely* | *Somewhat likely* | *Likely* | *Very likely* |

1. How likely are you to access a health educator through the phone to communicate your health concerns and improve your health? Why?

| **1** | **2** | **3** | **4** | **5** |
| --- | --- | --- | --- | --- |
| *Not very likely* | *Not likely* | *Somewhat likely* | *Likely* | *Very likely* |

**Intention to Use**

1. How open would you be to accessing a health educator through the phone or internet whenever you need help and advice?
2. How much more likely are you to access a health educator online or through your phone than going in-person?

| **1** | **2** | **3** | **4** | **5** |
| --- | --- | --- | --- | --- |
| *Not very likely* | *Not likely* | *Somewhat likely* | *Likely* | *Very likely* |

**Technology - Individual Interview Guide for High Blood Pressure Patients**

USE: This individual interview guide will seek out the thoughts and feelings of participants with high blood pressure (based on their BP measurements during an earlier home-based screening) on being referred to and using online telemedicine to provide them with health support. This survey uses the extended Unified Theory of Acceptance and Use of Technology (UTAUT2) model to guide the development of the following question guide.

Today, during this conversation, I would like to ask you a series of questions about how you use your telephone and the internet and your thoughts about receiving health care through either your phone, tablet, or your computer.

Demographic Questions

- May I please know how old you are?
- What do you identify as, male, female, or other?

mHealth Questions

1. Do you have a smartphone?

*yes no*

If participant answered no skip to question #6.

- 1. What do you use your phone for? (select all that apply)

|  | Texting |  |
| --- | --- | --- |
|  | Calling |  |
|  | Video calling |  |
|  | Taking/storing photos |  |
|  | Using the internet |  |
|  | Sending and receiving emails |  |
|  | Using apps |  |
|  | Watching videos |  |
|  | Other |  |

1. How much data do you feel you have monthly for your typical technology use?

| **1** | **2** | **3** | **4** | **5** |
| --- | --- | --- | --- | --- |
| *Not  enough data* |  |  |  | *More than enough  Data* |

1. How often do you use apps on your smartphone?

| **1** | **2** | **3** | **4** | **5** |
| --- | --- | --- | --- | --- |
| *Never* | *Once a day* | *Every couple  of days* | *Once a week* | *Multiple times a day* |

1. If the participant answered **never to #3,** *What are the reasons why you do not use apps? (select all that apply).*

|  | Don’t have the time |
| --- | --- |
|  | Too expensive |
|  | Don’t like apps |
|  | Don’t want to use apps |
|  | Don’t know how to use apps |
|  | Not enough data |
|  | Other (please specify): __________________ |

1. Have you ever used your phone to call a health clinic?

*yes no*

- 1. (If they answered **yes**) Why did you call the health clinic?

If NO, skip to #6. If YES, go to question b.

- 1. Was the call successful?

*yes no*

**Personal Experience**

1. Do you have any experience receiving health advice through your phone or the internet?

If NO, skip to the next section. If YES, go to question #7

1. Could you tell me a little about this experience?
   1. For what medical condition did you seek health advice through your phone or the internet?
   2. How long ago did you seek health advice through your phone or the internet?
   3. How would you describe your overall experience?
   4. Did you experience any technical difficulties that affected the quality of care you received from your telemedicine service?

**Geographical Location**

1. How strong is the internet connection where you live?

| **1** | **2** | **3** | **4** | **5** |
| --- | --- | --- | --- | --- |
| *Poor* |  | *Average* |  | *Excellent* |

1. How far is the nearest health clinic from where you live?
   1. How does the distance to the health clinic impact your decision to go there for health care?
   2. How might the distance to the health clinic impact your willingness to try telehealth (e.g., receiving health guidance through the telephone)?

**Data Privacy Issues**

1. How do you feel about talking to a health professional you do not know on your phone or through the internet?
2. How do you feel about the safety of the information you provide to a health professional through your phone or the internet?
   1. How might this impact your willingness to share your health information?

**Social Influence**

1. Do you know or have you heard of anyone who has received health care through their phone or the internet?

If NO, skip to the next section. If YES, continue to question #13.

1. a. What service did they use?

b. How did they describe their experience?

1. Do you know if there are any negative beliefs attached to using telemedicine? Could you please describe them?

**Effort Expectancy**

1. How do you feel about your ability and skills to use the internet?
2. How hard do you think using your phone to receive healthcare would be?
3. How do you feel about your ability to navigate through the internet to receive health services?
   1. If you had to schedule appointments, turn on your camera and audio and navigate through software to connect with a doctor or nurse what might be challenging for you?

**Hedonic Motivation**

1. How much comfort do you think you would experience from receiving healthcare through the internet?
   1. Probe further and ask how/why.
2. What level of comfort do think you would have speaking with a physician or nurse through the internet?

**Usage Behavior**

1. How likely are you to connect with health care professionals through the internet to communicate your health concerns? Why?

| **1** | **2** | **3** | **4** | **5** |
| --- | --- | --- | --- | --- |
| *Not very likely* | *Not likely* | *Somewhat likely* | *Likely* | *Very likely* |

1. How likely are you to connect with healthcare providers through the phone to communicate your health concerns? Why?

| **1** | **2** | **3** | **4** | **5** |
| --- | --- | --- | --- | --- |
| *Not very likely* | *Not likely* | *Somewhat likely* | *Likely* | *Very likely* |

**Intention to Use**

1. How open would you be to receiving health care through the phone or internet whenever you need medical care?
2. How much more likely are you to access health services online than in person?

| **1** | **2** | **3** | **4** | **5** |
| --- | --- | --- | --- | --- |
| *Not very likely* | *Not likely* | *Somewhat likely* | *Likely* | *Very likely* |
