# Supplementary material for: Exploring the Acceptability of Web-Based Health Modalities in Individuals With Hypertension: Qualitative Study
Source: J Med Internet Res. 2025 Aug 25;27:e72568. doi: 10.2196/72568 (PMC12377872; doi:10.2196/72568)
Supplement: Multimedia Appendix 2 [file jmir-v27-e72568-s002.docx]

| **Question** | **Type of Response** | **Number**  **of Cases** |
| --- | --- | --- |
| How much data do you feel you have monthly for your typical technology use?   \| **1** \| **2** \| **3** \| \| **4** \| \| **5** \| \| --- \| --- \| --- \| --- \| --- \| --- \| --- \| \| *More than*   *enough data* \| \| \|  \| \| *Not Enough*   *Data* \| \| \| | - Responded without using a numerical value - Did not provide a relevant response/ did not answer the question | 20  3 |
| How often do you use apps on your smartphone?   \| **1** \| **2** \| **3** \| **4** \| **5** \| \| --- \| --- \| --- \| --- \| --- \| \| *Never* \| *Once*  *a day* \| *Every couple*   *of days* \| *Once*  *a week* \| *Multiple times a day* \| | - Responded without using a numerical value - Did not provide a relevant response/ did not answer the question | 21  6 |
| How strong is the internet connection where you live?   \| **1** \| **2** \| **3** \| **4** \| **5** \| \| --- \| --- \| --- \| --- \| --- \| \| *Poor* \|  \| *Average* \|  \| *Excellent* \| | - Responded without using a numerical value - Did not provide a relevant response/ did not answer the question | 18  4 |
| How likely are you to access a health educator through the internet to communicate your health concerns and improve your health? Why?   \| **1** \| **2** \| **3** \| **4** \| **5** \| \| --- \| --- \| --- \| --- \| --- \| \| *Not very likely* \| *Not likely* \| *Somewhat likely* \| *Likely* \| *Very likely* \| | - Responded without using a numerical value - Did not provide a relevant response/ did not answer the question | 10  4 |
| How likely are you to access a health educator through the phone to communicate your health concerns and improve your health? Why?   \| **1** \| **2** \| **3** \| **4** \| **5** \| \| --- \| --- \| --- \| --- \| --- \| \| *Not very likely* \| *Not likely* \| *Somewhat likely* \| *Likely* \| *Very likely* \| | - Responded without using a numerical value - Did not provide a relevant response/ did not answer the question | 11  4 |
| How likely are you to connect with health care professionals through the internet to communicate your health concerns? Why?   \| **1** \| **2** \| **3** \| **4** \| **5** \| \| --- \| --- \| --- \| --- \| --- \| \| *Not very likely* \| *Not likely* \| *Somewhat likely* \| *Likely* \| *Very likely* \| | - Responded without using a numerical value - Did not provide a relevant response/ did not answer the question | 12  1 |
| How likely are you to connect with healthcare providers through the phone to communicate your health concerns? Why?   \| **1** \| **2** \| **3** \| **4** \| **5** \| \| --- \| --- \| --- \| --- \| --- \| \| *Not very likely* \| *Not likely* \| *Somewhat likely* \| *Likely* \| *Very likely* \| | - Responded without using a numerical value - Did not provide a relevant response/ did not answer the question | 15  1 |
| How much more likely are you to access a health educator online or through your phone than going in-person?   \| **1** \| **2** \| **3** \| **4** \| **5** \| \| --- \| --- \| --- \| --- \| --- \| \| *Not very likely* \| *Not likely* \| *Somewhat likely* \| *Likely* \| *Very likely* \| | - Responded without using a numerical value | 20 |
| How much more likely are you to access health services online than in person?   \| **1** \| **2** \| **3** \| **4** \| **5** \| \| --- \| --- \| --- \| --- \| --- \| \| *Not very likely* \| *Not likely* \| *Somewhat likely* \| *Likely* \| *Very likely* \| | - Responded without using a numerical value | 16 |

**Breakdown of non-numeric and irrelevant responses that were excluded from final analysis.**
